# Diagnosis and treatment of a rare bilateral primary thyroid cancer: a case report

**DOI:** 10.3389/fonc.2024.1468550

**Published:** 2025-02-13

**Authors:** Hai Lin, Xinyu Zhang, Na Yan, Tao Guo, Qiu Chen, Xianen Huang, Dandan Wang, Weili Wu

**Affiliations:** ^1^ Department of Endocrinology, The Third Affiliated Hospital of Wenzhou Medical University, Wenzhou, Zhejiang, China; ^2^ Wenzhou Medical University Renji College, Wenzhou, Zhejiang, China; ^3^ Key Laboratory of Digital Technology in Medical Diagnostics of Zhejiang Province, Hangzhou, Zhejiang, China; ^4^ Department of Traditional Chinese Medicine Orthopedics and Traumatology, The Third Affiliated Hospital of Wenzhou Medical University, Wenzhou, Zhejiang, China; ^5^ Department of Thyroid and Breast Surgery, The Third Affiliated Hospital of Wenzhou Medical University, Wenzhou, Zhejiang, China

**Keywords:** multiple thyroid nodules, PTC, MTC, molecular assisted diagnosis, case report

## Abstract

Preoperative ultrasound examination of thyroid nodules is the most economical and effective screening method for diagnosing thyroid nodules. Fine-needle aspiration biopsy (FNAB) cytology guided by ultrasound has high sensitivity and specificity in distinguishing benign and malignant thyroid nodules. However, approximately 25% of thyroid nodules cannot be determined by FNAB, and accurate differentiation of benign and malignant thyroid nodules is critical for patient prognosis. Here, we report the diagnosis and surgical treatment process of a rare patient with bilateral thyroid malignant tumor of independent origin. This patient had significantly elevated levels of calcitonin (Ctn: 130.00 pg/mL) and carcinoembryonic antigen (CEA: 16.13 ng/mL). Ultrasound shows a solid nodule on the left side of the thyroid gland, measuring 1.2*0.8*0.9cm, TI-RADS 4A; right solid nodule, 1.3*0.7*0.9 cm, TI-RADS 3. A fine needle biopsy of the left nodule showed little glandular epithelium and no evidence of malignancy. Multi-gene joint analysis of *RET C634R* in the left nodule and *BRAF V600E* in the right nodule indicated a potential diagnosis of left medullary thyroid carcinoma (MTC) and right papillary thyroid carcinoma (PTC). Postoperative pathology revealed the left thyroid nodule was MTC and the right nodule was PTC. The patient’s bilateral thyroid nodules are independent primary malignant lesions. This case emphasizes the important significance of combined analysis of ultrasound, serum biomarkers, cellular pathology, molecular detection, and paraffin pathology in the differential diagnosis of benign and malignant multiple thyroid nodules. It provides a reference for future diagnosis and treatment decisions of multiple thyroid nodules.

## Introduction

Thyroid nodules are a prevalent, frequently asymptomatic endocrine disorder diagnosed incidentally or upon physical examination. Ultrasonography is the primary method for the preliminary assessment of nodules. The incidence of thyroid nodules is about 20% - 76%, and malignant nodules are about 5% - 15% (1). Select nodules for FNAB cytology based on ultrasound characteristics, size, and high-risk clinical history. This procedure allows for a more precise diagnosis of the nodules’ benign or malignant nature through cellular pathology. FNAB cytology has become the “gold standard” for preoperative differentiation of benign and malignant thyroid nodules in domestic and foreign guidelines for diagnosing and treating thyroid nodules (2, 3). It is currently the most reliable method for diagnosing benign and malignant thyroid nodules. However, nodules whose nature cannot be determined by cytology still account for about 25%. At this time, genetic testing can further stratify the patient’s risk and determine whether the nodules are benign or malignant for observation or surgery (4). More than 90% of thyroid cancers are differentiated follicular cell-derived tumors, including papillary thyroid carcinoma (PTC) and follicular thyroid carcinoma (FTC). The rest include medullary thyroid carcinoma (MTC), which accounts for about 3-5% of thyroid cancers, as well as more rare thyroid anaplastic carcinoma (5, 6).

The concomitant presence of PTC and MTC is a very rare event in clinical practice, described in the literature mainly in case reports and a few studies. Poupak Fallahi et al. (7) reviewed 690 thyroid cancer patients diagnosed in a single center from 2001 to 2017 and found that only 5 cases, accounting for 0.72%, had both PTC and MTC present at the same time. MTC is a more invasive tumor than PTC. When MTC and PTC coexist, priority should be given to the management of MTC. If MTC is diagnosed early, a surgical cure can be expected. A multicenter study in Italy (8) collected data from 14 different treatment centers between 1992 and 2014, involving 183 patients with combined MTC/PTC. The study data showed that compared to PTC (stage IV: n=9, 4.92%), MTC (stage IV: n=27, 14.75%) has more patients diagnosed with stage IV. It was affect the patient’s prognosis. In a study evaluating the accuracy of preoperative diagnosis of MTC in multiple international centers (9), individual cytological assessment was found to have low sensitivity in MTC, limiting the ability for optimal preoperative assessment and initial surgery.

Here, we report the diagnosis and surgical treatment process of a rare patient with bilateral thyroid malignant tumor of independent origin. The patient has been diagnosed with bilateral thyroid nodules for over four years. Abnormal levels of Ctn and CEA were found during routine physical examinations. Ultrasound-guided biopsy was performed on the left 4a nodule, and a few glandular epitheliums were observed without evidence of malignancy. Multi-gene joint analysis showed the presence of *RET C634R* mutation in the left nodule. It is speculated that the left nodule may be MTC. Diagnostic surgery will be performed on the patient, and the proposed surgical approach is left thyroidectomy and isthmus resection (with partial or total right thyroidectomy and regional lymph node surgery). The intraoperative frozen section shows left MTC and right nodular goiter. In summary, the patient underwent total thyroidectomy and central lymph node dissection. Multi-gene joint analysis was performed on the postoperative tissue of the right nodule, which showed the detection of a BRAF V600E mutation in the driving gene, indicating that the right nodule is PTC. Postoperative pathology confirmed that the left thyroid nodule was stageIMTC and the right nodule was stageIPTC. The patient’s bilateral thyroid nodules are independent primary malignant lesions. The case underwent comprehensive preoperative evaluation through serum biomarker testing, ultrasound examination, cytological assessment, and multi gene testing combined assessment. Early diagnosis and initial surgical treatment were achieved for this case of MTC combined with PTC.

## Detailed case description

**Figure 1:**
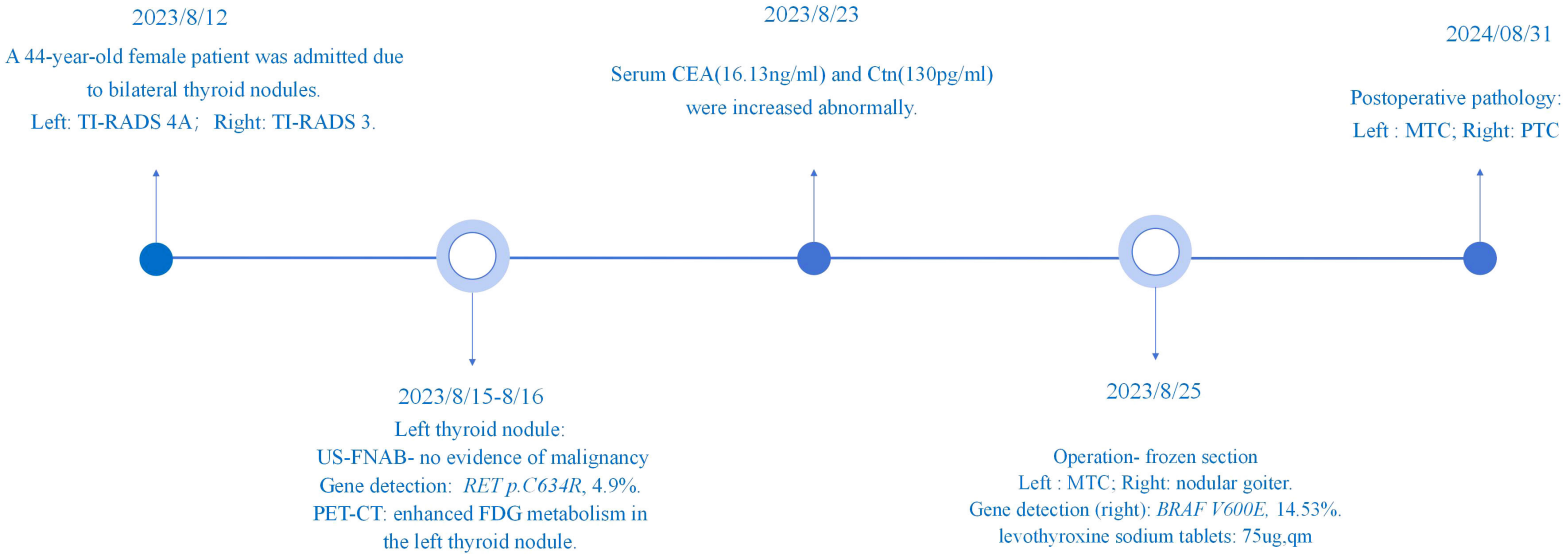
Display of patient diagnosis and treatment process. Timeline of patient’s diagnosis and treatment.

**Figure 2:**
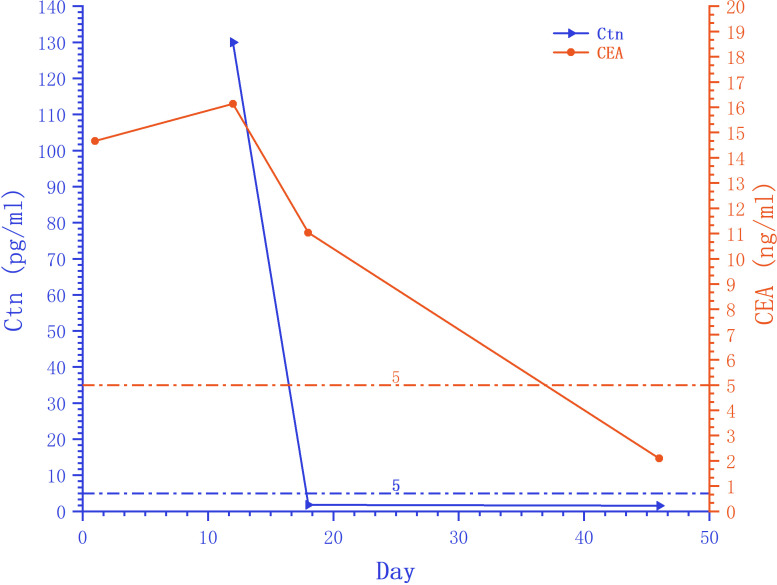
Trends in serum peripheral tumor marker Ctn and CEA. The serum peripheral tumor markers Ctn and CEA decreased to normal levels after surgery. The starting point of the horizontal axis is the patient’s admission time (August 12, 2023).

**Figure 3:**
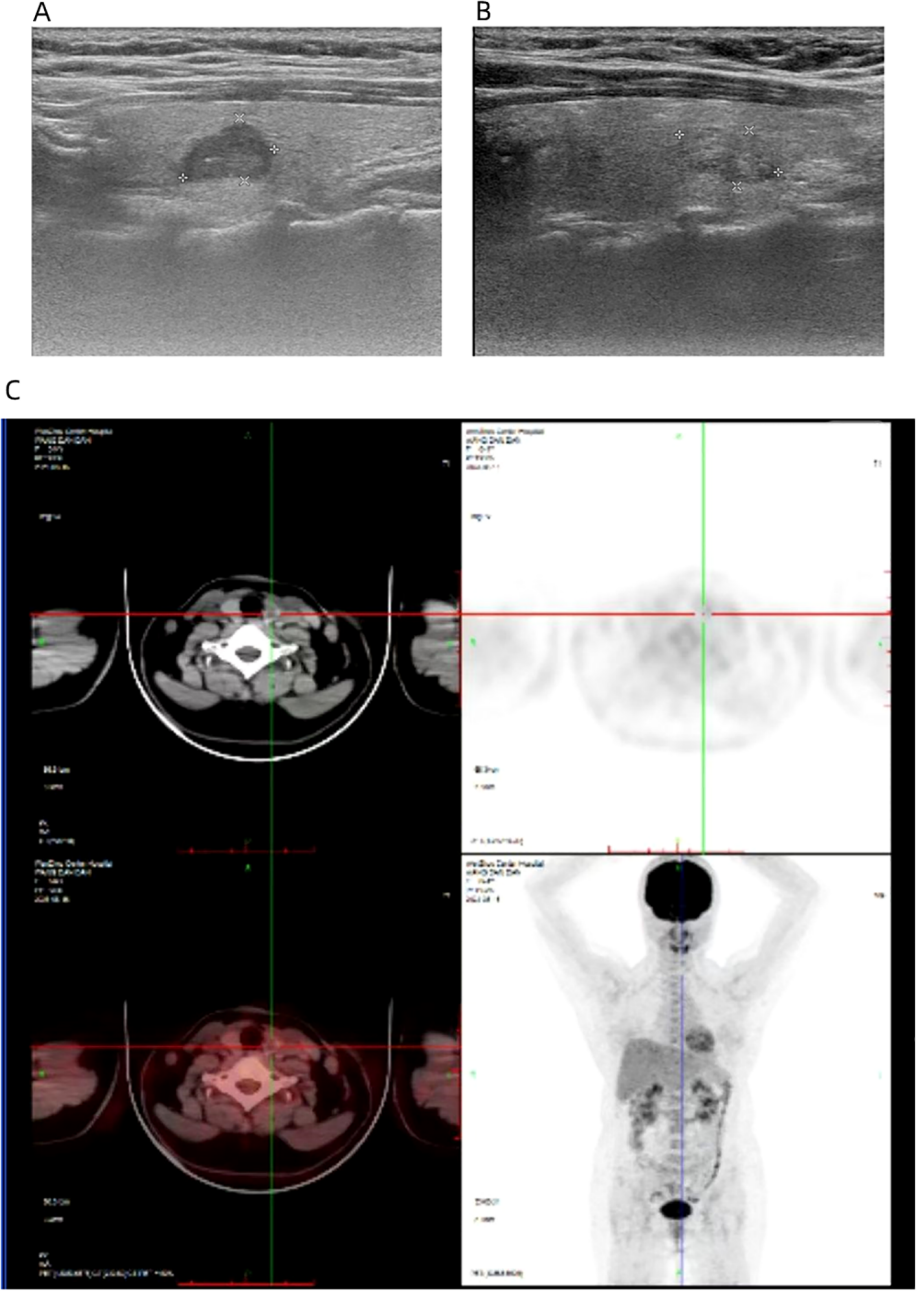
The ultrasound image shows a nodule of 1.2 * 0.8 * 0.9cm in the left thyroid gland **(A)** and a nodule of 1.3 * 0.7 * 0.9 cm in the right thyroid gland **(B)**. PET-CT revealed an increase in FDG metabolism in the left thyroid nodule, while whole-body imaging showed no abnormal increase in FDG metabolism **(C)**.

**Figure 4:**
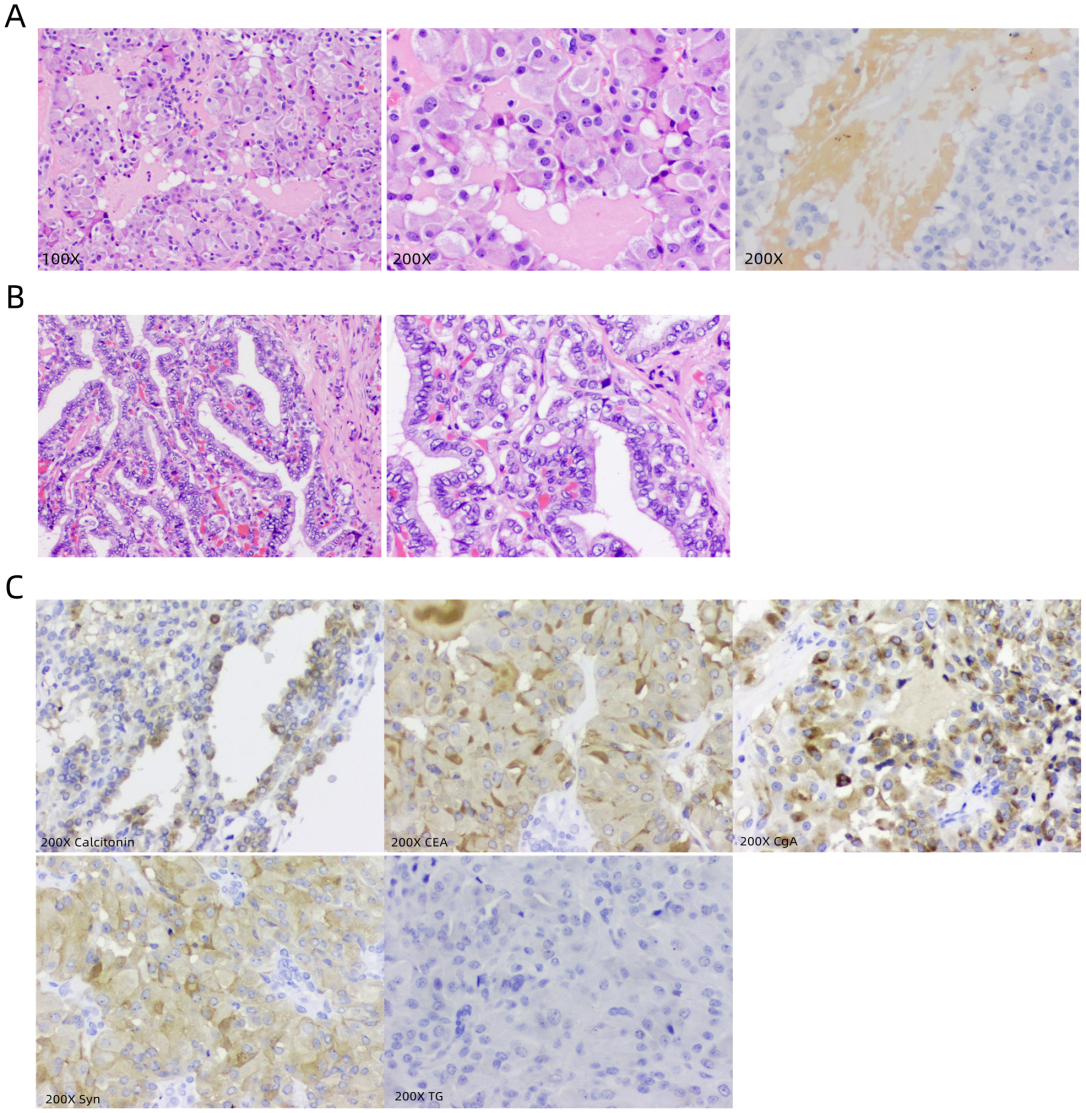
Presentation of postoperative pathological results. **(A)** Microscopic images of postoperative tissue sections of the left thyroid nodule, with 100X, 200X conventional staining and 200X congo red staining, respectively. **(B)** Microscopic images of postoperative tissue sections of the right thyroid nodule, with 100X and 200X conventional staining, respectively. **(C)** Immunohistochemical marker images under a 200X microscope are Calcitonin, CEA, CgA, Syn, and TG.

On August 12, 2023, a 44-year-old female patient was admitted due to the discovery of bilateral thyroid nodules during a physical examination for more than four years (**Figure 1**). The patient has no history of exposure to toxins or radioactive substances and no family history of thyroid tumors. The laboratory tests showed no abnormalities in Parathyroid Hormone (PTH), carbohydrate antigen199 (CA199), carbohydrate antigen 153 (CA153), carbohydrate antigen 125 (CA125), neuron-specific enolase (NSE), carbohydrate antigen 724 (CA724), alpha-feto protein (AFP), thyroid peroxidase (TPO), Thyroid Stimulating Hormone (TSH), Triiodothyronine (T3), Thyroxine (T4), Thyroglobulin (TG), Free Triiodothyronine (FT3), Free Tetraiodothyronine (FT4) and anti-thyroglobulin antibodies (TGAB). CEA:16.13 ng/ml (reference range < 5ng/ml), Ctn: 130.00 pg/mL (reference range < 5pg/mL), both abnormally elevated (**Figure 2**). Ultrasound examination showed a solid nodule on the left side of the thyroid gland with a size of 1.2 * 0.8 * 0.9cm, low echo, clear boundary, full shape, not smooth edge and a TI-RADS classification (10) of 4A (**Figure 3A**). It was recommended that malignant tumor be excluded from FNAB cytology. A solid nodule on the right side of the thyroid gland had a size of 1.3 * 0.7 * 0.9 cm, medium echo with weak echo, clear boundary, slightly full shape, smooth edge and was classified as Class 3 by TI-RADS, indicating a possible benign condition (**Figure 3B**). The PET-CT whole-body bone imaging results showed a slight increase in FDG metabolism in the left thyroid nodule (**Figure 3C**). An increase in FDG metabolism in the bilateral adnexal region (**Figure 3C**) was considered physiological uptake, and no other abnormal signs of FDG metabolism were observed. The puncture smear of the left lobe thyroid nodule under ultrasound guidance showed a small pile of glandular epithelium and no evidence of malignancy. At the same time, genetic testing is performed on the puncture cell fluid of the left lobe nodule of the thyroid gland to determine whether it is benign or malignant. The genetic testing showed a *RET p.C634R* somatic mutation in the left nodule and the mutation rate was 4.9%. Based on the PET-CT results, the FDG metabolism of the left thyroid nodule was increased, and laboratory Ctn and CEA were abnormally elevated. We speculate that the left thyroid nodule was likely to be sporadic medullary thyroid carcinoma (MTC). After sufficient communication and exchange with the patient, we decided to undergo surgical treatment and determine the nature of bilateral thyroid masses on August 25, 2023. During the operation, frozen tissue sections revealed ten lymph nodes in the “Left Zone III”, and no cancer metastasis has been detected yet. The left thyroid gland lobe and isthmus suggest epithelial-derived malignant tumor, considering medullary carcinoma. Intraoperative frozen sections showed nodular goiter in the right lobe of the thyroid gland. Based on the above intraoperative frozen section results, the diagnosis of bilateral thyroid masses was medullary carcinoma of the left thyroid and nodular goiter of the right thyroid. Therefore, the patient underwent total thyroidectomy with bilateral central lymph node dissection and left zone III lymph node biopsy. We also conducted molecular testing on the postoperative tissue of the right nodule while waiting for the paraffin results of the postoperative tissue. Postoperative paraffin tissue examination indicated that the left thyroid lobe was MTC (**Figure 4A**), it had invaded the surrounding thyroid gland, but there was no cancer invasion in the thyroid capsule, no cancer thrombus in the vasculature, and no cancer invasion in the nerve tissue. No metastases were identified in the left central lymph nodes, suggesting regional lymph node involvement was absent. Postoperative immunohistochemical markers showed: Calcitonin (+), TTF-1 (+), CgA (+), Syn (+), CEA (+), TG (-), Ki-67 (3%+), CK19 (-), CD56 (+), P53 (wild type) (**Figure 4C**). Surprisingly, residual right lobe thyroid nodules reported PTC (**Figure 4B**). Postoperative paraffin tissue examination showed PTC in the residual lobe of the right thyroid gland, with no evidence of neurovascular invasion. No metastasis of cancer was found in the lymph nodes of the right central region and the left third region. The *BRAF V600E* mutation was also reported by molecular examination of postoperative tissue from the right nodule and the mutation rate was 14.53%. Therefore, based on the comprehensive postoperative pathology and molecular testing results, the patient’s bilateral thyroid masses were ultimately diagnosed as T1N0M0 MTC of the left thyroid and T1N0M0 PTC of the right thyroid. The patient’s postoperative condition was stable and treated with oral administration of 75 ug, qm of levothyroxine sodium tablets to inhibit TSH (**Figure 1**). On August 29, 2023, the patient’s Ctn significantly decreased to 2pg/ml, and CEA was 11.03ng/ml, still not decreased to the normal range. On September 26, 2023, the patient’s Ctn remained below 2pg/ml, and CEA significantly decreased to 2.09ng/ml (**Figure 2**), returning to normal levels—instruct patients to follow up regularly in the later stage.

## Discussion

Thyroid nodules are a common disease and can be classified as benign and malignant nodules. Currently, ultrasound, MRI, serological examination, ultradense-guided fine needle aspiration biopsy cytology, and molecular diagnostic techniques are commonly used to determine the benign and malignant thyroid nodules before surgery (1, 11). However, the single detection method still has shortcomings, and the combined application of multi-means detection methods can improve the diagnostic accuracy of benign and malignant nodules. Ultrasound imaging plays an essential role in the diagnosis of thyroid nodules, and its accuracy rate in the diagnosis of thyroid cancer is as high as 90%. For nodules whose nature cannot be determined by ultrasound, Such as thyroid imaging reporting and data system (TI-RADS) grade 4 (12), ultrasound-guided fine needle aspiration biopsy is currently the most reliable method for diagnosing benign and malignant thyroid nodules (13). However, the ability to detect accurate results is limited by the operator’s level, nodule type, location/puncture needle type, bleeding during puncture, smear, etc., which limits the clinical application of US-FNAB cytology to a certain extent (14–16). Thyroid nodules were selected for FNAB cytology to obtain tissue for microscopic evaluation and cytological interpretation to diagnose the nature of the nodules definitively. The results of thyroid fine-needle aspiration cytology examination may yield uncertain results, and the tissue extracted by FNAB cytology can be utilized for molecular detection, providing further information on the risk of cancer (2). In current molecular testing, it is relatively clear that the BRAF V600E mutation is the most specific auxiliary diagnostic molecular marker for PTC (17). The 5th edition of WHO’s thyroid tumor classification highlights the role of molecular features in thyroid tumor classification (18). Malignant tumors of thyroid follicular cell origin are classified according to molecular characteristics and aggressiveness. PTC with multiple tissue subtypes is a BRAF-like malignancy. Invasive encapsulated follicular subtype PTC and thyroid follicular carcinoma are RAS-like malignancies. The origin cells of MTC are thyroid parafollicular cells, also known as C cells (19), which have the ability to secrete various peptides or active amine substances such as Ctn, calcitonin gene-associated peptides, and CEA. Therefore, MTC is a neuroendocrine tumor (20). The main cause of MTC is RET oncogene mutation. MTCs are divided into two categories: hereditary and sporadic. Almost all hereditary MTCs are accompanied by germline mutations in the *RET* gene, and 50% of sporadic MTCs have somatic mutations in the *RET* gene. The *RET* gene is also a therapeutic target for *RET*-driven malignant tumors (21), and targeted therapy is an important treatment method for advanced MTC with *RET* gene point mutations (22).

Although ultrasound examination is the preferred method for evaluating thyroid nodules and cervical lymph nodes, the existing thyroid imaging reporting and data system (TI-RADS) suggests that the malignant risk of thyroid nodules mainly targets papillary thyroid carcinoma (3). Therefore, the sensitivity and specificity of diagnosis for MTC are limited. FNAB, as a fast, economical, and safe method for distinguishing benign and malignant thyroid nodules, has been widely used in clinical practice. However, in a meta-analysis of 641 MTC patients included in 15 studies (23), the proportion of cases confirmed through FNAB was 56.4% (95% confidence interval, 52.6% to 60.1%), indicating that 40% of MTC may be missed based solely on FNAB results. Based on the characteristics of neuroendocrine tumors using MTC, detecting serum biomarker Ctn levels (individually or in combination with CEA testing) has been used for preoperative diagnosis (24). Serum Ctn is the main screening index of MTC. But serum Ctn levels can be influenced by various factors, such as smoking, drinking alcohol, taking drugs such as proton pump inhibitors, and suffering from certain diseases (such as C-cell proliferation, chronic kidney disease, small cell lung cancer, and gastrointestinal pancreatic neuroendocrine tumors) (25). Another serum biomarker, CEA, although associated with MTC invasiveness (26), is unsuitable for separate screening and diagnosis as it is not solely generated by MTC specificity (27). FNAB and serum Ctn are still insufficient in the diagnosis of MTC. Using FNA specimens to detect molecular changes related to MTC can be a supplementary diagnostic tool, especially for clinically suspected MTC, uncertain cytological results reported by FNAB, and those with serum Ctn in the gray area. Molecular diagnosis helps to identify MTC accurately, achieve early diagnosis and treatment (19).

In the case we reported, the patient was found to have bilateral thyroid nodules for over four years. Although abnormal increases in Ctn and CEA were observed in this patient. However, ultrasound and fine needle aspiration cytology did not clarify the nature of the nodule before surgery. A molecular examination of the puncture cell fluid from the left nodule revealed the RET somatic mutation p.C634R. We considered the potential limitations of a single diagnostic method, and after comprehensive complementarity of ultrasound, serum, FNAB, and molecular detection information, we considered the suspected medullary carcinoma of the left nodule. In the process of determining the nature of bilateral nodules during surgery, intraoperative frozen sections indicate that the left nodule is medullary carcinoma and the right nodule is nodular goiter. In order to improve the accuracy of judging the nature of nodules, we synchronously sent molecular detection for the right nodule. The molecular test results unexpectedly reported a *BRAF V600E* mutation, indicating that the right nodule is PTC. In the postoperative pathology of the right residual thyroid lobe, we confirmed that the right nodule is indeed papillary thyroid carcinoma.

The diagnosis and treatment process of bilateral thyroid nodules in this patient reflects the essential complementary role of ultrasound, serum, FNAB, molecular detection, and cell pathology combined detection in determining the nature of the nodules. It also reminds us that preoperative molecular testing for multiple nodules requires considering each lesion to determine the surgical plan in advance and avoid the occurrence of multiple surgeries.

## Data Availability

The original contributions presented in the study are included in the article/supplementary material, further inquiries can be directed to the corresponding author/s.
